# Single Positive Lymph Node Prostate Cancer Can Be Treated Surgically without Recurrence

**DOI:** 10.1371/journal.pone.0152391

**Published:** 2016-03-31

**Authors:** Dae Keun Kim, Kyo Chul Koo, Ali Abdel Raheem, Ki Hong Kim, Byung Ha Chung, Young Deuk Choi, Koon Ho Rha

**Affiliations:** 1 Department of Urology, CHA Seoul Station Medical Center, CHA University, CHA Medical School, Seoul, Republic of Korea; 2 Department of Urology, School of Medicine, Graduate School, Hanyang University, Seoul, Republic of Korea; 3 Department of Urology, Urological Science Institute, Yonsei University, College of Medicine, Seoul, Republic of Korea; 4 Department of Urology, Tanta University, Tanta, Egypt; 5 Department of Urology, Soonchunhyang University Cheonan Hospital, Cheonan, Chungcheongnam-do, Republic of Korea; National Health Research Institutes, TAIWAN

## Abstract

**Purpose/Objectives:**

To investigate pN1 prostate cancer (PCa) patients treated surgically without immediate adjuvant treatment.

**Materials and Methods:**

We analyzed the database of 2316 patients at our institution who underwent robot-assisted radical prostatectomy (RARP)/radical prostatectomy (RP) between July 2005 and November 2012. 87 patients with pN1 PCa and received no neoadjuvant and immediate adjuvant therapy were included in the study. Included pN1 PCa patients were followed up for median of 60 months. Biochemical recurrence (BCR)-free survival, metastasis-free survival (MFS), cancer specific survival (CSS), and overall survival (OS) rates were determined by using Kaplan-Meier analysis. Cox regression analysis was performed to investigate the impact of prostate-specific antigen (PSA) level, Gleason score, extraprostatic extension, seminal vesicle invasion, perineural invasion, lymphovascular invasion, positive surgical margin, tumor volume, early post-operative PSA(6 weeks), PSA nadir, lymph node yield, and number of pathologically positive lymph nodes on survival.

**Results:**

The 5-year OS rate of patients was 86.1%, while the CSS rate was 89.6%. The metastasis-free and BCR-free survival rates were 71% and 19.1%, respectively, and each was significantly correlated with the number of positive lymph nodes on log rank tests (p = 0.004 and p = 0.039, respectively). The presence of 2 or more pathologically positive LNs (HR:2.20; 95% CI 1.30–3.72; p = 0.003) and a Gleason score ≥8 (HR: 2.40;95% CI: 1.32–4.38; p = 0.04) were significant negative predictors of BCR free survival on multivariable regression analysis. Furthermore, the presence of 2 or more positive lymph nodes (HR: 1.06; 95% CI 1.01–1.11; p = 0.029) were significant negative predictors of metastasis-free survival on multivariable regression analysis. Additionally, in the patients who had no BCR without adjuvant treatment 9 patients out of 10 (90%) had single positive LN and 5 patients out of 10 (50%) had Gleason score 7. Therefore, single positive LN, and Gleason scores ≤7 have significantly low risk of disease progression.

**Conclusions:**

pN1 PCa patients have heterogenous clinical courses. Patients with single positive LN, and Gleason scores ≤7 have low risk of recurrence. Close observation with delayed adjuvant hormone therapy can be considered in these patients.

## Introduction

In prostate cancer (PCa) patients, the intraoperative diagnosis of lymph node (LN) metastasis had been lead to the abandonment of prostatectomy and had regarded as systemically disseminated disease associate with poor prognosis [1]. Other treatment such as external beam radiotherapy combined with systemic androgen deprivation treatment (ADT) was used for several decades. However, with emerging evidence, recent guidelines have recommended radical prostatectomy (RP) and robot-assisted radical prostatectomy (RARP) with extended pelvic LN dissection (ePLND) as a treatment modality in patients with high risk and very high risk PCa in the context of multimodal treatment [2, 3]. PCa with pathologically positive LNs (pN1) had been thought to have poorer prognosis than LN-negative PCa [4]. Nevertheless, pN1 PCa patients had variable long-term survival outcomes, and some patients with delayed postoperative treatment had no biochemical recurrence (BCR) or clinical progression, suggesting that immediate ADT is unnecessary in some pN1 PCa patients. We investigated pN1 PCa patients who diagnosed after RP/RARP with PLND but did not receive immediate adjuvant treatment. The aim of this study was to evaluate the possible factors that predict recurrence in patients with pN1 PCa.

## Materials and Methods

### Study patients

After obtaining institutional review board approval by human research protection center, severance hospital yonsei university health system (2014-0091-001), the patient records/information was anonymized and de-identified prior to analysis. We analyzed the data of 2316 patients at our institution who underwent RARP/ RP between July 2005 and November 2012. From this cohort, 124 (5.3%) patients with pN1 PCa with no distant metastases were identified. We excluded 17 patients who underwent neoadjuvant hormone treatment and 16 who underwent immediate adjuvant hormone treatment and 4 patients who underwent immediate adjuvant radiation therapy. Thus, 87 patients were ultimately included in the study.

All the patients were preoperatively evaluated by using chest radiography, abdominal/pelvic computed tomography, prostate magnetic resonance imaging, and whole body bone scanning according to their physicians’ discretion. BCR was defined as prostate serum antigen (PSA) levels >0.2 ng/mL with secondary confirmatory increase at least 6 weeks after surgery.

Patients with low and intermediate risk localized PCa and life expectancy over 10 year were decided to perform RARP/RP. Furthermore, patients with selected high risk and very high risk localized PCa were decided to perform RARP/RP in the context of multimodality treatment. Patients underwent RARP/RP with PLND and surgery was carried out by three surgeons.

### Clinical assessment

RARP was performed using a transperitoneal approach. Decision to perform ePLND was based on the risk of lymph node metastases.The high risk and very high risk prostate cancer patients who have probability of lymph node invasion over 4% underwent ePLND, and the intermediate risk prostate cancer patients have underwent standard PLND (sPLND) [5, 6]. The boundaries of sPLND included the the external iliac and obturator LNs, whereas the boundaries of ePLND additionally included the internal iliac, presacral, and common iliac LNs up to the ureteric crossing [7]. All LN specimens were serially sectioned at 3 mm, fixed in 10% neutral buffered formalin, and embedded in paraffin blocks. Each cut was stained with hematoxylin and eosin, and examined microscopically for the presence of cancer cells by a single genitourinary pathologist with >15 years of experience.

In our study patients didn’t receive immediate adjuvant treatment either ADT or radiotherapy. Currently, the indication of ADT or radiotherapy on pN1 PCa was not established. Some center recommended cut off value of PSA > 5ng/mL for ADT or radiotherapy [8]. In this study, the indication of ADT or radiotherapy was PSA > 2ng/mL or appearance of significant symptoms (Bone or visceral metastasis, pain, hydronephrosis, bladder outlet obstruction, or gross hematuria).

All patients included in this study had complete clinical and pathological data available, including age, pre-operative PSA, early post-operative PSA (6 weeks), clinical and pathological stages (according to the 2002 American Joint Committee on Cancer staging system), pathological Gleason score, surgical margin status, tumor volume, the number of LNs removed, and the number of positive LNs. Patients were followed-up with physical examinations and PSA measurements every 6 weeks~3 months during the first year after surgery, every 6 months during the second year, and annually thereafter.

### Statistical analysis

Kaplan-Meier analysis was performed to investigate BCR-free survival, metastasis-free survival (MFS), cancer specific survival (CSS), and overall survival (OS) rates after surgery for a median follow-up period of 60 months. Location of recurrence and time to recurrence were analyzed. Cause and time of death were identified from death certificates and medical records in the database of the National Cancer Registry Center. Univariable and multivariable Cox proportional regression analyses were performed to investigate the predictive factors for BCR and metastasis after RARP/RP.

Continuous variables were presented as median and interquartile range (IQR) values. The log-rank test was employed to evaluate subgroup survival rates. Cox proportional regression analyses were performed to derive the predictive factors for BCR and metastasis after RARP/RP. A two-tailed p value ≤0.05 was deemed statistically significant. Analysis was performed using the SPSS v.20.0 software (SPSS Inc., Chicago, IL, USA).

## Results

### Baseline characteristics

Table 1 shows the clinical and pathological characteristics of the total cohort and pN1 PCa patients. In this cohort, the median follow-up time was 60 months (IQR 49–69). The median age at surgery was 67 years (IQR 62–72). Of these patients, 78 patients (89.7%) had locally advanced PCa, 67 patients (77%) had concomitant extraprostatic extension, and 51 (58.6%) had seminal vesicle invasion. Only 1 patient (1.1%) had a Gleason score of 6 in our cohort, whereas a majority of 68 patients (78.1%) had Gleason scores of 8–10. From total 87 pN1 patients, 19 patients (21.8%) had clinically positive lymphadenopathy on preoperative image. The median number of LNs removed was 21 (IQR 16–29). Of the 87 patients, 35 patients (40.2%) had 1 positive LN, 20 patients (24.1%) had 2 positive LN, and 32 patients (36.8%) had 3 or more pathologically positive LNs. The highest number of positive LNs in a patient was 18. The positive surgical margin rate was 65.5% in our cohort.

**Table 1:** Clinical characteristics of total cohort and patients and pN1 prostate cancer after radical prostatectomy/robot-assisted radical prostatectomy.

Characteristics	Total Cohort, n = 2316	pN1 Patients, n = 87
Age, year, median (IQR)	65 (60–70)	67 (62–72)
BMI, kg/m^2^ (IQR)	24.12 (22.49–25.73)	24.33 (22.1–25.65)
ASA score category (%)		
1	1413 (61)	44 (50.6)
2	871 (37.6)	42 (48.3)
3	32 (1.4)	1 (1.1)
Preoperative PSA ng/mL, median (IQR)	7.4 (5.1–12.3)	15.91 (9.18–37.91)
Early post-operative PSA ng/mL, median (IQR), 6weeks	0.01 (0.01–0.02)	0.05 (0.02–0.11)
Prostate volume, cc (IQR)	36.8 (28.2–48.4)	34 (27.2–43.3)
Tumor volume, cc (IQR)	2.5 (1–4)	6.5 (3–15)
Clinical T stage (%)		
T1	810 (35)	5 (5.7)
T2	950 (41)	9 (10.3)
T3a	348 (15)	21 (24.1)
T3b	185 (8)	43 (49.4)
T4	23 (1)	9 (10.3)
Clinical N stage (%)		
N0	2224 (96)	68 (78.2)
N1	92 (4)	19 (21.8)
Pathologic T stage (%)		
T2	1413 (61)	9 (10.3)
T3a	718 (31)	27 (31)
T3b	162 (7)	44 (50.6)
T4	23 (1)	7 (8.1)
Pathologic Gleason score (%)6	695 (30)	1 (1.1)
7	1135 (49)	18 (20.8)
8	255 (11)	20 (23)
9	224 (9.7)	45 (51.7)
10	7 (0.3)	3 (3.4)
Extraprostatic extension (%)	853 (36.8)	67 (77)
Seminal vesicle invasion (%)	185 (8)	51 (58.6)
Positive surgical margin (%)	797 (34.4)	57 (65.5)
PSA nadir (ng/mL), median (IQR)	0.01 (0.01–0.02)	0.03 (0.01–0.26)
Time to PSA nadir (month),median (IQR)	2 (1–3)	2 (1–3)
Lymph node yield, median (IQR)		21 (16–29)
Number of positive lymph node (%)		
Mean (median)		3.07 (2)
Range		1–18
1		35 (40.2)
2		20 (24.1)
3		13 (14.9)
4		5 (5.7)
≥5		14 (16.1)

ASA = American Society of Anesthesiologists; PSA = prostate-specific antigen; IQR = interquartile range.

### Oncological outcomes

The 5-year OS rate of the patients was 86.1% (95% confidence interval [CI], 81–95) and the CSS rate in the was 89.6% (95% CI, 83–97). The 5-year MFS and BCR-free survival rates were 71% (95% CI, 74–88) and 19.1% (95% CI, 18–31), respectively. The patients without BCR neither clinical progression was detailed in Table 2. From 16 patients of BCR-free, 10 patients (11.5%) had no adjuvant treatment for the total follow up period. The 10 patients with BCR-free and no adjuvant treatment patients had single positive lymph node except for just one patient. Clinical stage N0 was found in 9 patients and only 1 patient had cN1. For Gleason score distribution, Gleason 7 was 4 patients, for Gleason 8 was 4 patients, and Gleason 9 for 2 patients.

**Table 2:** Characteristics of pN1 PCa patients with no immediate recurrence.

Patient	Age, year	Preoperative PSA ng/mL	Clinical TNM stage	Pathologic Gleason score	Extraprostatic extension	Seminal vesicle invasion	Surgical margin	PLND type	Lymph node yield	Number of positive lymph node	Adjuvant treatment
1	64	52.9	T3aN0M0	4+4	(+)	(+)	(-)	ePLND	21	2	(-)
2	69	10.04	T3aN0M0	4+3	(+)	(-)	(-)	ePLND	18	2	ADT 24 month
3	73	17.12	T3aN0M0	4+3	(+)	(-)	(+)	ePLND	29	1	(-)
4	69	32.63	T2cN0M0	4+3	(+)	(-)	(-)	sPLND	16	1	(-)
5	68	7.04	T3aN0M0	4+4	(+)	(+)	(-)	sPLND	19	1	(-)
6	59	6.21	T2bN0M0	3+4	(+)	(+)	(-)	ePLND	28	1	(-)
7	69	22.2	T3bN0M0	3+4	(+)	(-)	(-)	ePLND	47	1	ADT 42 month
8	69	5.68	T3aN0M0	4+3	(+)	(-)	(+)	ePLND	25	2	RT 36month ADT 60 month
9	55	19.71	T2cN0M0	5+5	(-)	(-)	(-)	ePLND	19	1	RT,ADT 36 month
10	67	37.91	T3bN0M0	5+4	(-)	(-)	(+)	ePLND	24	3	ADT 36 month
11	62	11.89	T3aN0M0	4+5	(-)	(+)	(+)	ePLND	15	1	(-)
12	78	6.39	T1cN0M0	4+3	(-)	(-)	(-)	sPLND	12	1	(-)
13	81	76.43	T3aN0M0	4+3	(+)	(-)	(-)	ePLND	19	2	ADT 24month
14	58	6.79	T3bN0M0	4+3	(-)	(+)	(+)	ePLND	28	1	(-)
15	68	9.87	T3bN1M0	5+4	(-)	(+)	(+)	sPLND	11	1	(-)
16	69	13.81	T3bN0M0	4+4	(+)	(-)	(-)	ePLND	25	1	(-)

ADT = androgen deprivation treatment; RT = radiation treatment; sPLND = standard pelvic lymph node dissection; ePLND = extended pelvic lymph node dissection.

After median follow up of 60 months, in single positive lymph node patients, 3 patients died due to prostate cancer, 6 patients had clinical progression of metastasis, and 20 patients had BCR and 16 patients remained with no recurrence (biochemical and clinical). However, in 2 or over 2 lymph node positive patients, 9 patients had died due to prostate cancer, 22 patients had clinical progression of metastasis, and 44 patients had asymptomatic PSA increase and just 8 patients remained with no recurrence (biochemical and clinical).

Overall 16 patients have expired. Four patients have been dead not related to prostate cancer, related with ischemic heart disease, COPD, and other malignancy. Of these 4 patients, 1 patient was disease free, 1 patient had only BCR, and other 2 patients had clinical progression with bone metastasis.

**Fig 1:**
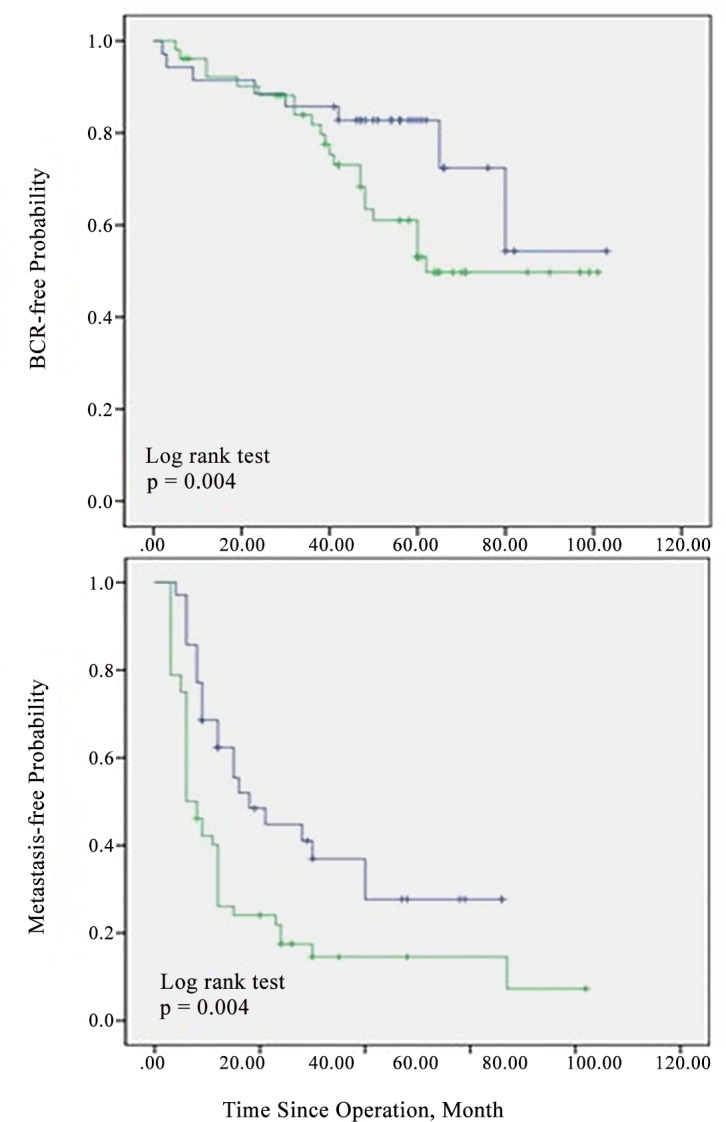
Kaplan-Meier curve for biochemical recurrence-free survival and metastasis-free survival by the number of positive lymph nodes. Blue line = one positive lymph node; green line = two or more positive lymph nodes.

There was a significant difference between each of the BCR-free rates, MFS and the number of positive LNs (One positive LN vs two or more positive LN) as determined by log rank tests (p = 0.004 and p = 0.039, respectively) (Fig 1). The BCR-free survival rate was also significantly higher in patients with Gleason scores ≤7 than in those with Gleason scores ≥8 (20.7% vs. 14.3% respectively, p = 0.005).

Nineteen patients (21.8%) had local recurrence in the prostatic bed, 14 patients (16.1%) had recurrence in pelvic lymph node. From patients of either local recurrence or pelvic lymph node recurrence, 25 patients had recurrence with bone or visceral metastases. Only 4 patients had exclusive local recurrence and no exclusive recurrence in pelvic lymph node. From the total clinical recurrence patients, the 25 patients (78.1%) had bone or concomitant visceral metastasis (3 patients of lung metastasis, 3 patients of liver metastasis). From total 25 metastasis patients, 17 patients (68%) were still alive after distant metastasis.

The presence of 2 or more pathologically positive LNs (HR: 2.20; 95% CI 1.30–3.72; p = 0.003) and a Gleason score ≥8 (HR: 2.40; 95% CI: 1.32–4.38; p = 0.04) were significant negative predictors of BCR-free survival on Cox proportional hazard multivariable regression analysis (Table 3). Furthermore, the presence of 2 or more positive lymph nodes (HR: 1.06; 95% CI 1.01–1.11; p = 0.029) were significant negative predictors of metastasis-free survival on Cox proportional hazard multivariable regression analysis (Table 4). Table 5 shows the contemporary reports on oncologic results of pN1 PCa. In the patients who had no BCR without adjuvant treatment, 9 patients out of 10 (90%) had single positive LN and 5 patients out of 10 (50%) had Gleason score 7.

**Table 3:** Cox proportional hazard regression analysis of predictive factors associated with biochemical recurrence after surgical treatment with pN1 prostate cancer patients.

Biochemical recurrence	Univariable (unadjusted HR)			Multivariable (adjusted HR)		
Predictive factor	HR	95% CI	*P*- value	HR	95% CI	*P*- value
PSA	1.00	0.99–1.01	0.968			
Pathologic Gleason score ≥8	2.19	1.21–3.97	0.01	2.40	1.32–4.38	0.004
Lymphovascular invasion	1.08	0.64–1.82	0.77			
Perineural invasion	1.88	0.92–3.84	0.081	1.06	0.60–1.87	0.835
Extracapsular invasion	1.26	0.67–2.37	0.463			
Seminal vesicle invasion	1.35	0.81–2.23	0.248			
Positive surgical margin	1.51	0.88–2.57	0.135			
Tumor volume	1.02	0.99–1.05	0.317			
PSA nadir	1.02	0.91–1.13	0.79			
Early PSA persistence PSA≥0.1 ng/ml at 6 weeks after surgery	1.10	0.60–2.01	0.76			
Lymph node yield	1.00	0.97–1.02	0.852			
Positive lymph node, no.						
1 (reference)	Ref	Ref	Ref	Ref	Ref	Ref
≥2	1.94	1.15–3.26	0.012	2.20	1.30–3.72	0.003

HR = hazard ratio; CI = confidence interval; PSA = prostate-specific antigen; ref = reference.

**Table 4:** Cox proportional hazard regression analysis of predictive factors associated with distant metastasis after surgical treatment with pN1 prostate cancer patients.

Distant metastasis	Univariable (unadjusted HR)			Multivariable (adjusted HR)		
Predictive factor	HR	95% CI	*P*- value	HR	95% CI	*P*- value
PSA	1.01	0.99–1.02	0.728			
Pathologic Gleason score ≥8	1.05	0.31–3.56	0.934			
Lymphovascular invasion	1.82	0.82–4.08	0.094	1.93	0.86–4.37	0.115
Perineural invasion	0.58	0.17–1.98	0.384			
Extracapsular invasion	1.49	0.56–3.97	0.426			
Seminal vesicle invasion	0.94	0.44–2.01	0.875			
Positive surgical margin	1.27	0.54–2.99	0.587			
Tumor volume	1.04	0.98–1.10	0.206			
PSA nadir	0.94	0.69–1.29	0.716			
Early PSA persistence PSA≥0.1 ng/ml at 6 weeks after surgery	0.83	0.34–2.07	0.831			
Lymph node yield	0.77	0.31–1.93	0.576			
Positive lymph node, no.						
1 (reference)	Ref	Ref	Ref	Ref	Ref	Ref
≥2	1.05	1.01–1.10	0.034	1.06	1.01–1.11	0.029

HR = hazard ratio; CI = confidence interval; PSA = prostate-specific antigen; Ref = reference.

**Table 5:** Comparison of contemporary reports on oncologic results of pN1 prostate cancer.

Series	Year	Method of operation	pN1 cases, n	Median follow up (months)	LN yield Median (IQR)	Survival
Spiess et al.[8]	2007	RP	100	62	11 (3–32)	5yr BCRFS 50%; 5yr PFS 84%; 10yr PFS 69%; 5yr CSS 94%; 10yr CSS 75%
Touijer et al.[9]	2014	RP	369	48	15 (10–21)	5yr BCRFS 54%; 10yr BCRFS 35%; 5yr PFS 79%; 10yr PFS 65%; 5yr CSS 94%; 10yr CSS 72%
Seiler et al.[10]	2014	RP	88	187	21 (6–41)	10yr BCRFS 8%; 10yr PFS 26%; 10yr CSS58%
Nini et al.[12]	2015	RP	800	76	19 (13–26)	5yr BCRFS 53.7%; 5yr PFS 50.5%; 5yr CSS 58.8%
Moschini et al.[13]	2015	RP	1011	211	13 (9–18)	15yr BCRFS 34.1%; 15yr PFS 67%; 15yr CSS 20%
Current study	2016	RP/RARP	87	60	21 (16–29)	5yr BCRFS 19.1%; 5yr PFS 71%; 5yr CSS 89.6%

BCRFS = Biochemical recurrence rate free survival; PFS = Progression free survival; CSS = Cancer-specific survival; OS = Overall survival; RP = Radical prostatectomy; RARP = Robot-assisted radical prostatectomy.

## Discussion

In this study, we investigated the role of RP/RARP with PLND in pN1 PCa patients by determining the oncological outcomes as assessed by BCR and MFS rates in pure Asian cohort. Included patients in our study had relatively higher Gleason score PCa compared with American and European researches [9, 10]. Patients of pN1 PCa who underwent RP/RARP with PLND and without adjuvant treatment had a 19.1% chance of remaining BCR-free during the 5-year follow up, as well as a 71% chance of remaining metastasis-free. Similar with western researches, the number of positive LNs and Gleason scores were significantly related with BCR-free survival, MFS rate.

In previous study, Boorjian et al. reported on the long term RP outcome of pN1 PCa. The 10-yr CSS was 85.8% with 89.7% of patients receiving adjuvant ADT [4]. In another study of pN1 PCa patients who did not receive adjuvant ADT, the 7-yr BCR-free rate was 10.9% [11]. The clinical course of pN1 PCa is not all lethal and it is heterogeneous. It can be associated with no clinical progression even in the absence of adjuvant treatment. We have found that ≥2 positive LNs significantly increase the risk of BCR and metastasis, as does a Gleason score ≥8 in BCR. Thus, the clinical outcomes vary in pN1 PCa patient populations according to the positive LN burden and the Gleason score. On detail analysis of 10 patients who were surgically cured without BCR and had no adjuvant therapy, only 1 patient had 2 positive LNs. While the other 9 patients had single positive LN. The patients with microscopic metastatic deposit of LN such as this single positive LN patients had a chance for no adjuvant treatment. The number of diseased lymph node of ≥2 had deleterious impact on BCR and metastasis.

Few studies were investigated to assess the efficacy of no adjuvant treatment in patients with pN1 PCa after surgical treatment. Touijer et al.[9] have investigated 369 pN1 PCa patients who underwent RP and ePLND with no adjuvant treatment at a median follow up of 48 months. The predicted 10-yr BCR free survival, and MFS rates were 28%, and 65%, respectively. The 10-yr OS, and CSS rates were 60%, and 72%, respectively. Predictors of BCR risk were Gleason score >7, positive surgical margin and ≥3 positive LNs. Additionally, Seiler et al. [10] have investigated the long-term oncological result of 88 patients with pN1 PCa who underwent RP and PLND with a median follow up of 15.6 years. The number of positive nodes was the significant prognostic factor for CSS since patients with ≥2positive nodes had a threefold greater risk of cancer specific death. The 10-yr OS and CSS were 51%, 58%, respectively. Comparing the oncologic outcome between 1 positive LN group and ≥2positive LN group, incidence of BCR free survival was 18% in 1 positive LN group, 54% of patients showed clinical progression and 31% died of PCa. However, ≥2 positive LN group revealed 0% of BCR free survival within 10 years, only 10% had clinically progression-free survival, and two-thirds of patients have died of PCa.

In terms of clinical recurrence pattern on pN1 PCa, Nini et al [12] have investigated 800 pN1 PCa who underwent RP and ePLND with a median follow-up of 76 months. One-third of patients with pN1 PCa experienced clinical recurrence and from those patients, one-third had local or nodal recurrence. Experiencing local or nodal recurrence had higher 5-yr CSS rates compared with those of reptroperitoneal nodal, skeletal, and visceral recurrence. The site of recurrence (skeletal, visceral), pathologic grade≥pT3b, pathologic Glesason score 9–10 were independent predictor of CSS. Moschini et al.[13] investigated the natural history of 1011 pN1 PCa patients with a median follow-up of 211 months. Nearly all patients received adjuvant ADT and adjuvant radiation treatment (aRT) was given based on patient discretion. The 15-yr clinical recurrence rate was 33%. The solitary locations were skeletal (55%), nodal (34%), local soft tissue (17%), and visceral (5%). Predictors for clinical recurrence were Gleason score 8–10, number of positive nodes, and more recent year of surgery. The 15yr CSS after clinical recurrence was 20%. Multiple recurrences, skeletal, and visceral metastases were significantly associated with CSS. (Table 5)

In our study, the clinical recurrence with no immediate adjuvant treatment cohort, only 4 patients (4.6%) had exclusive pelvic local recurrence, and no patient had exclusive pelvic LN recurrence. Furthermore, eventually 25 patients (28.7%) had bone or concomitant visceral metastases. The patients who showed clinical progression in pN1 PCa, had higher rate of distant metastasis. Therefore, systemic ADT rather than local radiation therapy could be considered in the setting of adjuvant therapy for higher burden positive LNs

According to current guidelines, ePLND is recommended in patients with a high risk of LN metastasis [2]. Recently, the use of RARP as sharply risen while PLND have been decreased, even in patients with high-risk PCa, because of the technically challenging and time consuming aspects of the procedure, especially with the advent of robotics [14]. However, for intermediate- and high-risk PCa patients, PLND is critical for tumor control and survival due to the fact that it eliminates the chance of micrometastases.

Positive LNs after RP were uncommon and decreased over the last 30-years with the incidence of 8.3%, 3.5%, and 1.4% based on pre, early, and contemporary PSA eras [15]. Since the recent introduction of robotics, the clinical course after RARP in pN1 PCa has not been investigated [16]. BCR and metastasis after surgical treatment of pN1 PCa remains an issue of concern in robotic era, even though not all patients with pN1 PCa develop BCR or metastasis.

In the past, pN1 PCa was considered a poor prognostic factor associated with a limited chance of cure and poor long-term survival regardless of treatment modality. Although the multimodal approaches involving surgical, radiological, and hormone treatments have shown to be beneficial. However, the optimal standard management has not been clearly established yet. The majority of patients with pN1 PCa receive ADT after surgery, however, the indication and timing of ADT vary among clinicians. Several studies have investigated the survival benefit of surgical management of pN1 PCa over hormone treatment alone [17]. Messing et al. concluded that immediate adjuvant ADT after RP and PLND improved the survival of pN1 PCa patients, since 77% of patients who received immediate ADT were alive and had no evidence of recurrent disease, including undetectable serum PSA levels compared to 18% of patients who did not receive ADT after sugery[18].

As some pN1 PCa patients experienced long-term BCR-free and progression-free status, accurate identification of those patients with pN1 PCa who will benefit from immediate ADT is critical. Furthermore, evidence suggests that the timing of ADT should be adapted for each individual according to the risk of clinical progression, as the optimal timing and indication of adjuvant ADT in pN1 PCa is still a matter of debate [18].

Patients with low LN burdens (single positive LN), and a low Gleason scores (≤7) could be able to defer immediate adjuvant ADT because of the low risk of BCR and clinical progression. Spiess et al. suggested that ADT can be delayed until BCR, indicated by PSA >5 ng/mL, without impairing the oncologic outcome [8]. Delaying adjuvant ADT in selected pN1 PCa patients has some advantages. First, it restricts adjuvant ADT only to high risk patients of clinical progression after surgery. Second, it avoids related side effect such as hot flashes, osteoporosis, and muscle loss. Third, it prevents overtreatment and saves treatment costs. Therefore, immediate ADT should not be routinely offered to patients who have a minimal metastatic burden. It seems that patients with increased number of positive LNs may be benefit from extended templates, while patients with a lower positive LN burden may have better prognosis. Moreover, the PCa with Gleason scores ≤7 has low aggressiveness, and less clinical progression.

In our study, we had a median follow up of 60 months which was not enough for evaluation of the long-term clinical recurrence course or death due to the new introduction of robotics system. However, we could evaluate recent era by including pN1 PCa cases who underwent both of RP and RARP. In addition, we investigated individual aspects of cases with neither clinical recurrence nor BCR, and the majority of cases revealed single positive LN. We excluded patients who received neoadjuvant or immediate adjuvant therapy. Therefore, we could select and predict with no recurred cases of pN1 PCa.

Our study contains certain limitations. First, it is a retrospective study, and thus contains inherent selection biases towards patients who underwent a particular surgical technique. Second, the median follow-up period of 60 months for our cohort was short to assess the long-term recurrence patterns and survival rates for PCa in robotic era. Third, the PLND template varied according to individual patient`s LN invasion risk and surgeon preference, however, median LN yield was 21 which was comparable with ePLND cases. Despite these limitations, the novelty of our study is clear in the fact that it is the first Asian oncological investigation and individual analysis for identification of pN1 PCa patients who could be treated by surgery alone without the need of immediate adjuvant therapy.

## Conclusions

Patients with single positive LN and Gleason scores ≤7 have chance of no recurrence after surgical treatment. Close observation with delayed adjuvant hormone therapy can be considered in those patients. Additionally, the majority of pN1 PCa who revealed clinical progression had distant metastasis. Therefore, systemic adjuvant ADT could be considered with patients who have high LN burden ≥2.

## Abbreviations

ADT: Androgen deprivation therapy
LN: Lymph node
PCa: Prostate cancer
PSA: Prostate serum antigen
PLND: Pelvic lymph node dissection
ePLND: Extended pelvic lymph node dissection
RP: Radical prostatectomy
RARP: Robot-assisted radical prostatectomy
OS: Overall survival
CSS: Cancer specific survival
